# Acquired Senile Retinoschisis of the Peripheral Retina Imaged by Spectral Domain Optical Coherent Tomography

**DOI:** 10.7759/cureus.14540

**Published:** 2021-04-18

**Authors:** Rahaf A Mandura

**Affiliations:** 1 Department of Ophthalmology, King Abdulaziz University, Jeddah, SAU

**Keywords:** acquired senile retinoschisis, spectral domain optical coherent tomography, sd-oct, retinal detachment, argon laser photocoagulation

## Abstract

Senile retinoschisis (SR) is a rare eye disease characterized by the abnormal separation of the neurosensory retina layers typically at the outer plexiform layer. Retinal detachment (RD) can be associated with SR in approximately 0.05% of the cases in which urgent treatments are indicated. The utility of spectral domain optical coherent tomography (SD-OCT) is helpful in diagnosing SR and distinguishing it from RD. This is a case of a 63-year-old man who presented with right eye floaters for a duration of two months. There were no other optic symptoms such as flashes of light, decreased vision, or pain. The best-corrected visual acuity was 20/25 in both eyes. Dilated fundus examination of the right eye revealed two elevated dome-shaped, mobile, transparent, smooth, and round peripheral retinal lesions in the inferotemporal quadrant suggestive of SR. SD-OCT was utilized to diagnose SR and rule out RD. In conclusion, SD-OCT is a very valuable diagnostic tool that can be utilized for SR which is a rare condition that can have serious visual consequences if not diagnosed and managed properly.

## Introduction

Senile retinoschisis (SR), either acquired or degenerative, is a rare and hard-to-diagnose eye disease characterized by the abnormal separation of the neurosensory retina layers due to microcystic degeneration, typically at the outer plexiform layer [1]. The reason why it is difficult to diagnose is that it is mostly asymptomatic and can be undetected for all of the life unless it is discovered incidentally or a serious complication occurs [2]. It was first described in 1933 by Bartels and was explored in derails in 1985 by Byer [3, 4]. In most cases, it is an asymptomatic bilateral disease with a prevalence rate between 1.65% and 7.00% among individuals above 40 years of age affecting the peripheral retina in most of the cases [5]. Acquired retinoschisis affects men and women equally and is not known to be linked genetically [1]. This differs from congenital retinoschisis, which is an X-linked recessively inherited vitreoretinal degeneration characterized by splitting in the nerve fiber layer typically in the fovea [6].

Progressive and symptomatic retinal detachment (RD) associated with SR is rare, occurring in approximately 0.05% of the cases [4]. It is the only complication in which urgent treatment is indicated and is often difficult to conclusively differentiate between localized RD and retinoschisis using clinical observation alone [7]. The utility of spectral domain optical coherent tomography (SD-OCT) in successfully distinguishing retinoschisis from RD and revealing a more detailed picture of the retinal morphology has been established [8]. This is a case of SR in the peripheral retina in which SD-OCT was utilized to report the retinal finding.

## Case presentation

A 63-year-old man presented to the ophthalmic outpatient clinic complaining of floaters in the right eye (OD) for a duration of two months. There were no flashes of light, decreased vision, pain, history of trauma, or previous ocular disease. The medical history was significant only for bronchial asthma with albuterol inhalers as the sole medication used while the past surgical history reported a gastric sleeve operation which was done five years ago.

**Figure 1:**
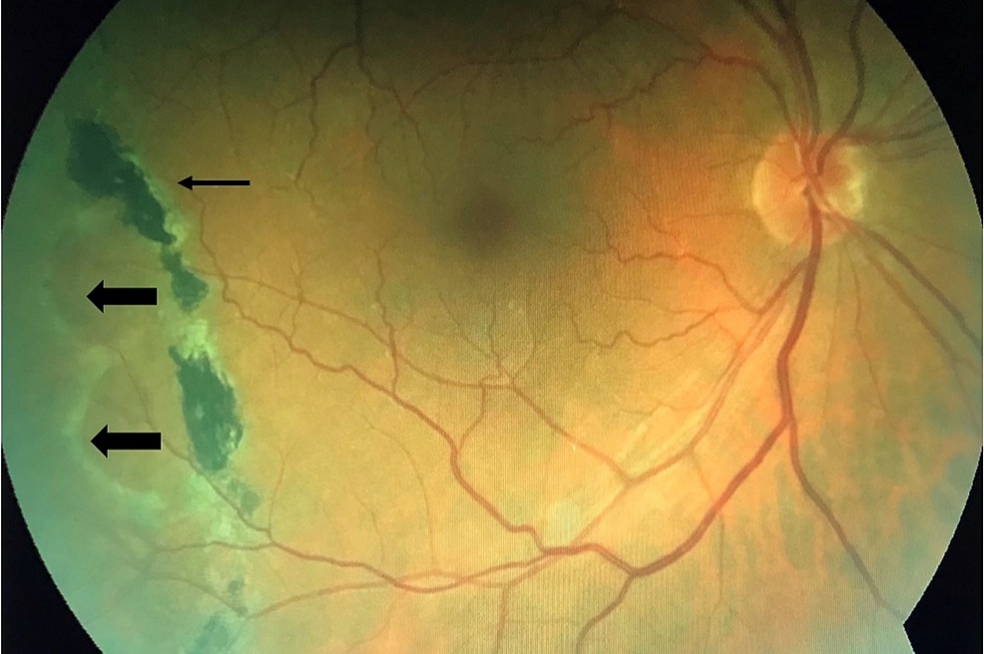
Fundus photo of the right eye showing senile retinoschisis at the inferotemporal quadrant (thick arrows), and barrage argon laser photocoagulation scar (thin arrow).

**Figure 2:**
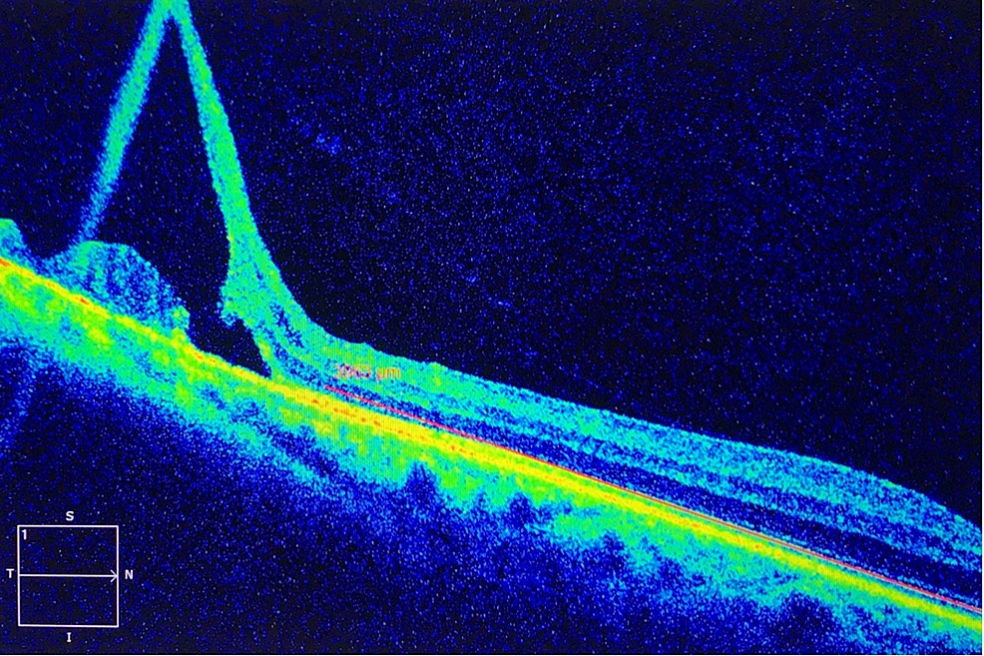
Spectral domain optical coherent tomography showing senile retinoschisis.

On examination, the best-corrected visual acuity was 20/25 OD and 20/25 in the left eye (OS). Intraocular pressure was measured by air-puff tonometer and showed 17 mmHg OD and 16 mmHg OS. Meanwhile, no relative afferent pupillary defect was noted. Slit-lamp anterior segment examination of both eyes was normal except for mild nuclear sclerosis cataracts. Dilated fundus examination of the right eye revealed two elevated dome-shaped, mobile, transparent, smooth, and round peripheral retinal lesions in the inferotemporal quadrant. The lesions were extending from 7:30 to 9 meridians and posteriorly with an estimated distance of one and a half-disc diameters (Figure 1). It was uncertain whether the fluid shifted when the patient changed position or not. However, there were no hemorrhage, exudate, pigment changes, or demarcation lines. Scleral indentation revealed no obvious break or hole. The macula was normal and the remainder of the retinal examination was unremarkable. Afterward, dilated fundus examination of the left eye was normal. On the other hand, B-scan ultrasonography of the right eye demonstrated dome-shaped elevations with high intensity while A-scan ultrasonography showed echo spikes. Therefore, SD-OCT was performed to rule out localized peripheral RD. SD-OCT was performed at the lesion and demonstrated attached retina with a wide separation of the neurosensory retina with splitting found at the outer plexiform layer, characteristic of SR (Figure 2). For treatment, a barrage argon laser photocoagulation was performed to surround the retinoschisis and prevent further extension (Figure 1). The patient was followed up regularly and the long-term follow-up after four years showed stable findings without any progression.

## Discussion

SD-OCT is increasingly favored over conventional time-domain OCT for the evaluation of a variety of retinal disorders due to its higher resolution imaging and easier interpretation [1]. Faster acquisition times allow more images to be acquired with fewer motion artifacts. This feature is especially important for obtaining images of peripheral retinal pathologies which requires the patient to fixate at the extremity of their gaze [1]. In this case, SD-OCT provided detailed documentation of the microanatomic structural changes in the retinal periphery, which confirmed the diagnosis of SR, allowing appropriate and successful management of the condition. The enhanced resolution provided information, supporting the earlier observation that abnormal splitting of the neurosensory retina occurs typically at the outer plexiform layer [9].

This case presented with symptomatic unilateral SR in the inferotemporal quadrant which is uncommon. Most patients are asymptomatic and this is an incidental finding without ocular or visual complaints. Although the location of SR in the inferotemporal quadrant was found to be more common than other quadrants [4, 5], the unilateral presentation is rare and unusual. Even though SR is usually benign, vision-threatening complications can occur including a posterior extension of the SR cavity, outer wall breaks and schisis detachment, and progressive rhegmatogenous retinal detachment (RRD). Schisis detachment occurs when schisis fluid accumulates in the subretinal space while RRD occurs when both the inner and outer layers break allowing liquefied vitreous to gain access to the subretinal space [10].

In this case, the patient was treated with a barrage argon laser photocoagulation that created a posterior pigmented demarcation photocoagulation scar which prevented the posterior extension of SR and macular involvement. This goes in line with Yassur et al. [11] results as they found that the 57 eyes with SR that were treated with argon laser photocoagulation had a partial or complete collapse of SR and none of them progressed after treatment which proves its efficacy as an uncomplicated outpatient procedure in halting the progression of SR or development of RD [11].

## Conclusions

SD-OCT is an instrumental tool that is utilized to diagnose SR which is a rare condition that can have serious visual consequences if not diagnosed and managed properly. In addition, early laser photocoagulation treatment is considered a safe uncomplicated simple outpatient procedure that is beneficial in preventing the possible complications of long-standing retinoschisis.
